# Case of Superfœtation

**Published:** 1846-10

**Authors:** 


*Case of Superfœtation.* By M. Mounier.—Catharine Fournier Lafond, aged 32 years, tall, thin, and well formed; the catamenia have always been irregular. In June, 1845, they ceased to appear, and certain disorders she experienced induced her to think herself pregnant. At the end of August, the menses reappeared on two occasions at an interval of fifteen days, but their continuance was short, and quantity small. She vomited somewhat also at this time. The signs becoming more marked, and the catamenia having ceased permanently, she again returned to her former opinion. In fact, on the 28th of February, pains commenced, which continued all the following day and night. Towards morning, on the 2d of March, they appeared to diminish. The neck of the uterus was greatly dilated, the waters were slowly discharged, and the uterus became very sluggish in its activity. The child presented in a good position. Two grammes of ergot of rye were administered, under the influence of which the pains returned. A few contractions sufficed to produce the birth of a dead child, which was well formed, and had all the appearances of a fœtus at the full time. The placenta was not long in being expelled. About five o'clock in the evening, nine hours after the accouchement, without great pain, or any effort, she experienced the sensation of a body passing through the vagina. She thought that some organs of the abdomen were protruding, and was considerably frightened. The nurse (*sage-femme*) was recalled in haste. On raising the sheets, she found a second fœtus, having all the characters of one from four and a half to five months old, with a cord, a placenta still bloody, membranous envelopes, all perfectly intact and well preserved. Both were of the female sex.

Cases of superfœtation are so surprising that their existence has been denied by the greater number of authors. The supposition is only admissible, according to them, in individuals who have a double uterus. Foderè, however, has not adopted this opinion, and recently practitioners of good faith have cited authentic examples of it. Such are those which Dr. Levret of Lyons, communicated to the Academy in 1842, where fœtuses perfectly developed were expelled with two, three, and four months intervening between them. Such also are those recorded by Dr. Chervin, relative to negresses in Guadaloupe. One of these gave birth to a black and then a white infant, and another to a black and then a mulatto child, at an interval of some months from each other. The case reported by M. Mounier is singular, from the physiological condition observed in the woman. The menses appeared twice in August, giving rise in her mind to a supposition contrary to the truth. The second fecundation must have taken place in the month following this period, that is, during September. The menses then ceased permanently to appear. The two beings enjoying a life in common, grew and were developed separately, and in this manner arrived at the term of parturition for the first. This occurred at the period first calculated, that is, nine months after the first cessation of the catamenia. The term of the second would have also been nine months, had not the labor, and perhaps the effect caused by the administration of the ergot of rye, induced in it conditions which terminated fatally, and caused its expulsion.—*Monthly Jour. of Med. Science, from Gazette Medico-Chirurgicale.*

